# Laparoscopic appendectomy for appendiceal intussusception assisted by colonoscopy: A case report

**DOI:** 10.1016/j.ijscr.2021.106611

**Published:** 2021-11-18

**Authors:** Kei Ohira, Takeshi Ohki, Yuji Inoue, Masakazu Yamamoto

**Affiliations:** Institute of Gastroenterology, Tokyo Women's Medical University, 8-1, Kawada-cho, Shinjuku, Tokyo, Japan

**Keywords:** Appendiceal intussusception, Colonoscopy, Laparoscopy, Appendectomy, Malignancy, Case report

## Abstract

**Introduction:**

Appendiceal intussusception sometimes results from appendiceal cancer. Ileocecal resection instead of appendectomy is often chosen as a treatment as it is technically difficult to resect the appendix alone without causing dissemination of appendiceal cells to the abdominal cavity. Herein, we present the first report of a case in which appendiceal intussusception was treated by resection of the appendix alone via simultaneous colonoscopy and laparoscopy.

**Presentation of case:**

A 40-year-old man underwent laparoscopic appendectomy for appendiceal intussusception. Since a neoplastic cause could not be completely ruled out, we planned to carry out oncologically safe appendectomy that would not expose the tumor to the abdominal cavity. The resection was performed in the lumen of the cecum rather than in the abdominal cavity to prevent dissemination of appendiceal cells to the peritoneal cavity during surgery. Histopathologic examination revealed chronic inflammation of the appendix but no malignancy. The postoperative course was uneventful.

**Discussion:**

Here, we present a case in which laparoscopic resection of the mesoappendix and colonoscopy-assisted resection of the appendix were performed in combination in a patient with appendiceal intussusception. When the possibility of malignancy is low, this maneuver may prevent patients from undergoing ileocecal resection with lymph node dissection as it prevents exposure of the abdominal cavity to the tumor.

**Conclusion:**

Simultaneous performance of laparoscopy and intraoperative colonoscopy is feasible and, from an oncological viewpoint, may be preferable when the cause of appendiceal intussusception is unknown or malignancy is not suspected.

## Highlights

• Appendiceal intussusception with unknown cause usually requires ileocecal resection.
• We successfully treated this disorder via combination of colonoscopy and laparoscopy.
• Our procedure is advantageous in cases with incidental malignancy.

## 1 Introduction

Appendiceal intussusception is a rare disease, with a prevalence of 0.004 to 0.01% [1]. If malignant findings are suspected from preoperative imaging, laparoscopic ileocecal resection is the procedure of choice. If a malignant tumor is not suspected as the cause of appendiceal intussusception, a laparoscopic appendectomy is often performed, and if the pathology results show any malignant findings, an additional ileocecal resection with lymph node dissection is performed. In this way, a two-stage surgery may be necessary. However, it is technically difficult to perform an appendectomy for appendiceal intussusception with the appendix completely inverted. Here, we report a case of appendiceal intussusception that was treated via laparoscopic mesoappendectomy and colonoscopic appendectomy in a single operation.

This case report has been reported in line with the SCARE Criteria [2].

## 2 Presentation of case

A 40-year-old man presented at a local hospital with stomachache. He underwent colonoscopy and was diagnosed with appendiceal intussusception. He was referred to our hospital for further examination. He had no past medical history or any relevant family history. He had never smoked and was a social drinker.

**Fig. 1:**
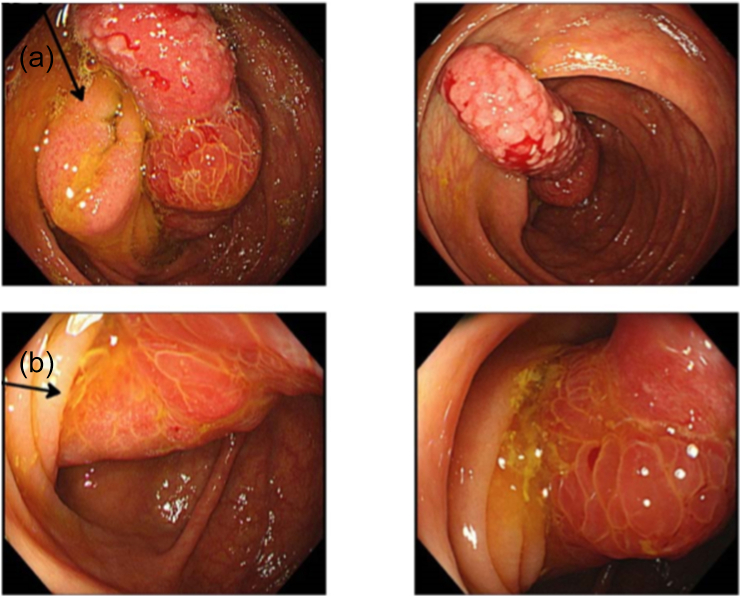
Colonoscopy showed a swollen appendix with redness which was inverted to the ascending colon. Arrow (a) and (b) showed ileocecal valve and appendiceal orifice respectively.

**Fig. 2:**
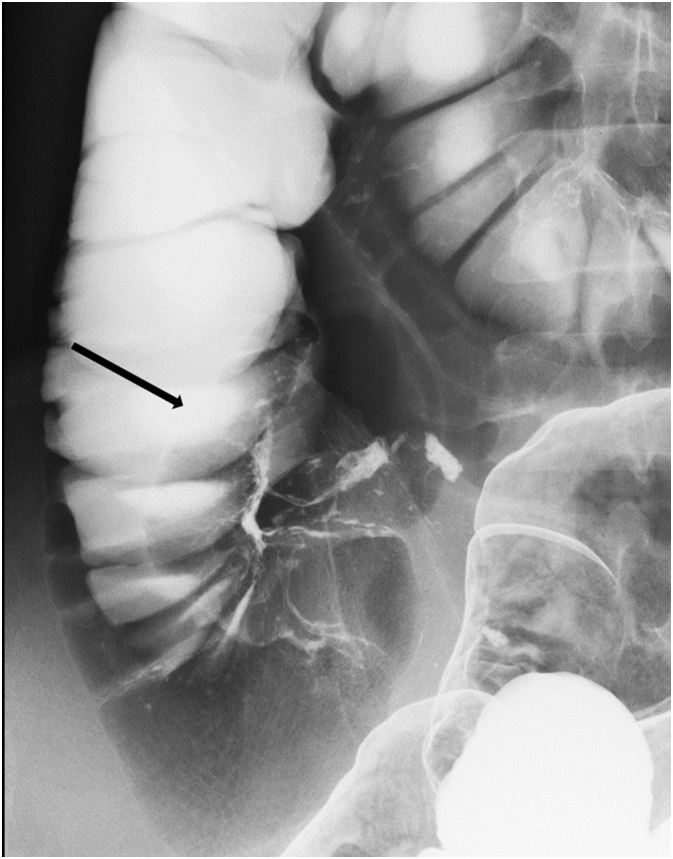
Gastrografin enema showed translucency shaped with swollen appendix (arrow).

**Fig. 3:**
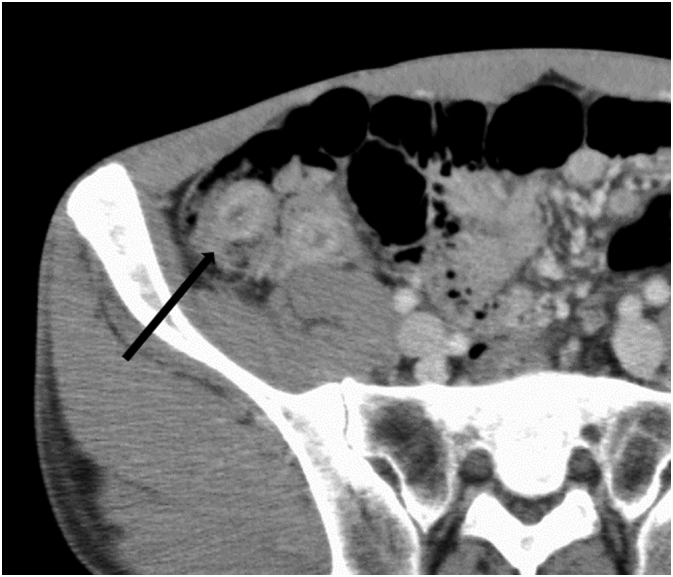
Abdominal enhanced CT showed intussusception of appendix and no tumor on top of it (arrow).

On admission, his body temperature, blood pressure, heart rate, and respiratory rate were 36.7 °C, 111/62Hg, 69 bpm, and 16 breaths/min, respectively. His abdomen was soft and flat without tenderness. Neither the white blood cell count nor the C-reactive protein level was elevated, and tumor marker levels were within normal limits on laboratory tests. Colonoscopy showed a swollen appendix with redness, which was inverted to the ascending colon (Fig. 1). There were no elevated lesions on the appendix or irregular patterns in its mucosa. Contrast enema showed translucency shaped with swollen appendix (Fig. 2). The proximal portion of the appendix was located in the cecum and was completely inverted. Enhanced computed tomography of the abdomen showed intussusception of the appendix with no tumors (Fig. 3). No swollen lymph nodes were seen in the ileocecal mesentery. The cause of the intussusception was not identified in the preoperative examinations, which meant that malignancy could not be ruled out.

**Fig. 4:**
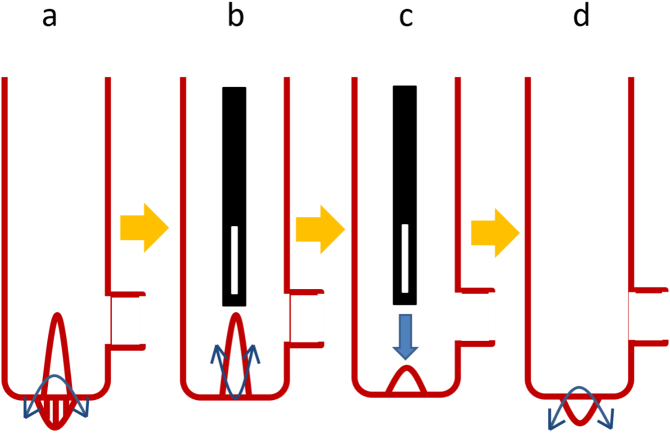
a) Incision of the mesoappendix by laparoscopic maneuver. b) Resection of the appendix by colonoscopy snare. c) Reduction of intussusception of the rest of the appendix by colonoscopy. d) Resection of the rest of the appendix by laparoscopic maneuver.

Based on the diagnosis of appendiceal intussusception, we performed colonoscopy-assisted appendectomy at the same time as laparoscopic mesoappendix resection to prevent dissemination of appendiceal cells to the abdominal cavity during the resection. On laparoscopy, the appendix was seen to be totally invaginated to the cecum. After ultrasonic coagulation of the appendiceal artery using a laparoscopic maneuver (Fig. 4a), we resected the inverted appendix by performing an intraoperative colonoscopy with a bipolar snare (Fig. 4b); the colonoscopic resection line was made close to the appendiceal orifice, aiming for total resection. Then, the resected appendix was retrieved using an endoscopic pouch. Finally, we resected the root of the appendix (which could not be resected via colonoscopy) with a laparoscopic linear stapler after pushing it with colonoscopy forceps to achieve its reduction (Fig. 4c, d). The root was retrieved using a laparoscopic pouch.

**Fig. 5:**
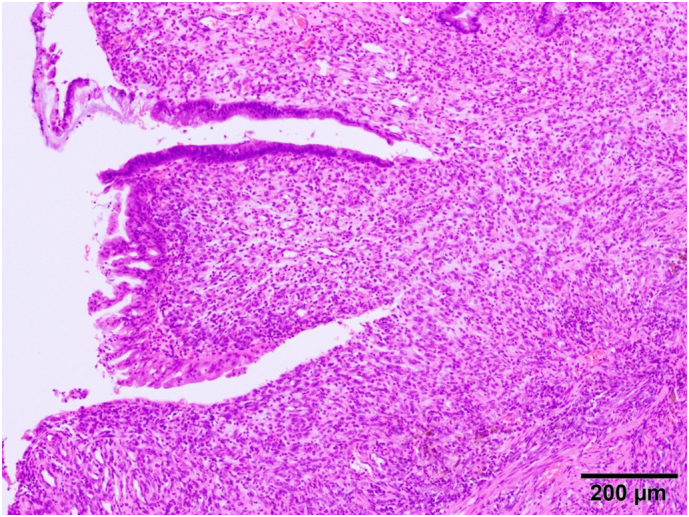
Histopathological examination showed inflammatory granulation on extracted appendix and no malignant findings.

Histopathology examination showed inflammatory granulation on the extracted appendix but no evidence of malignancy (Fig. 5). The appendiceal intussusception was caused by chronic appendicitis. The patient was discharged, and his post-operative course was uneventful.

## 3 Discussion

**Fig. 6:**
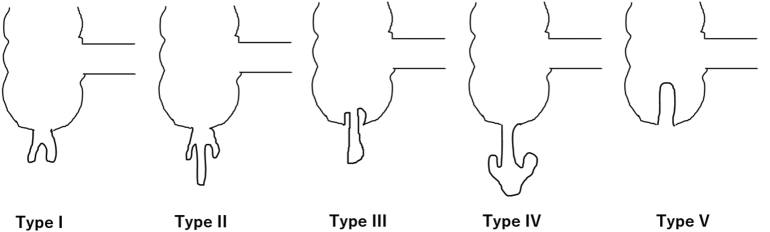
Type I: The tip of the appendix is inverted into itself. Type II: The tip of the appendix intrudes into itself without inversion. Type III: The proximal portion of the appendix intrudes into the cecum. Type IV: The proximal portion of the appendix intrudes into the distal portion of it. Type V: The whole appendix is inverted into the cecum.

Appendiceal intussusception is thought to be rare. A PubMed search from 1999 to 2020 using “appendiceal intussusception and laparoscopic appendectomy” as the search term retrieved only 11 case reports. Appendiceal intussusception was first reported by McKidd in 1858 [3] and was classified by McSwain in 1941 [4], Fink in 1964 (Fig. 6) [5], and Atkinson in 1976 [6]. The present case is classified as Type V as per McSwain; in Type V, the entire appendix invaginates into the cecum, while in Type I to IV, invagination of the appendix is partial.

Intussusception of the appendix may result from abnormal peristalsis [5]. The causes of appendiceal intussusception in the 11 cases identified in our literature search were as follows: endometriosis (three cases) [7], [8], [9], lymphoma (two cases) [10], [11], adenoma (two cases) [12], [13], and hyperplasia [14], mucocele [15], torsion [16] and pregnancy (one case each) [17]. Appendiceal intussusception is diagnosed via ultrasonography, computed tomography, contrast enema, and colonoscopy. In our case, colonoscopy showed polyp-like invagination of the appendix in the cecum, with no other features of note. Characteristic radiologic findings for appendiceal intussusception include the coiled spring sign [17], [18] and the finger-like defect [19]. Contrast enema showed the finger-like defect in our case, which is consistent with Type V appendiceal intussusception as classified by McSwain [4] and Fink [5] (Fig. 6).

In the case reports cited above, laparoscopic appendectomy was an effective treatment for appendiceal intussusception. However, the appendiceal stump might cause the invagination to recur [14]. In our case, not only laparoscopy, but also colonoscopy, aided the removal of the stump. Performance of appendectomy using mainly colonoscopy helps ensure the completeness of the resection and prevents the dissemination of possibly malignant appendiceal cells. Simultaneous colonoscopic and laparoscopic maneuvers for treatment of appendiceal intussusception have not been reported in the literature to date. From an oncological standpoint, our procedure has the advantage of preventing recurrence. Recently, combination of laparoscopic and endoscopic surgery has also been performed. Gastric GISTs generally do not require lymph node dissection and laparoscopic endoscopic cooperative surgery (LECS) developed by Hiki et al. preserves as much function as possible with minimal partial resection of the stomach [20]. With a similar concept, the present case was proposed to avoid ileocecal resection in a minimally invasive manner and to safely resect the appendix.

The cause of the appendiceal intussusception in our case was not identified preoperatively. Hence, exposure of the appendix to the peritoneal cavity during laparoscopy was unfavorable from an oncological viewpoint. Our procedure minimized the invasiveness of the surgery by avoiding the need for colectomy. Combination of performance of colonoscopy and laparoscopy for appendiceal intussusception could be an advantageous option in cases with suspected or confirmed malignancy. This method could be an option for minimally invasive surgery for Type V appendiceal intussusception.

## 4 Conclusion

We reported a case of appendiceal intussusception caused by chronic appendicitis. Endoscopic appendectomy and coincident laparoscopy were feasible, and the best treatment option as the cause of the intussusception was not determined preoperatively. This method could prevent dissemination of appendiceal cells to the abdominal cavity and oncologically preferable just in case of incidental malignancy.

## Provenance and peer review

Not commissioned, externally peer-reviewed.

## Ethical approval

This report is not a research study.

## Funding

None.

## Guarantor

Kei Ohira.

## Research registration number

Not applicable.

## CRediT authorship contribution statement

Kei Ohira wrote the manuscript and assisted the endoscopic maneuver.

Takeshi Ohki performed laparoscopic appendectomy.

Yuji Inoue performed endoscopic maneuver.

Masakazu Yamamoto was engaged in perioperative management.

## Declaration of competing interest

The authors have no conflicts of interest to declare.
